# Identification of long non-coding RNAs biomarkers associated with progression of endometrial carcinoma and patient outcomes

**DOI:** 10.18632/oncotarget.17537

**Published:** 2017-04-30

**Authors:** Yanan Sun, Xiaoyan Zou, Jun He, Yuqin Mao

**Affiliations:** ^1^ Department of Gynecology and Obstetrics, Daqing Oilfield General Hospital, Daqing 163000, China

**Keywords:** biomarkers, endometrial carcinoma, long non-coding RNA

## Abstract

Endometrial carcinoma is a complex disease characterized by both genetic, epigenetic and environmental factors. Increasing evidence has suggested that long non-coding RNAs (lncRNAs) play important roles in the development and progression of cancers. In this study, we performed a comparison analysis for lncRNA expression between patients with early-stage (stage I/II) and those with advanced-stage (stage III/IV) derived from The Cancer Genome Atlas (TCGA) project and identified 17 differentially expressed lncRNAs using student t-test. Five of the 17 differentially expressed lncRNAs were selected as optimal biomarkers that are significantly associated with progression of UCEC using random forest feature selection procedure. A risk classifier of five lncRNAs was developed to as a molecular signature that identifies patients at high risk for progression using support vector machine. Results of five-lncRNA risk classifier achieved high discriminatory performance in distinguishing advanced stage from early stage with 78% prediction accuracy, 96.6% sensitivity and 76.6% specificity. Functional analysis suggested that these five lncRNA biomarkers may play critical roles in the progression of UCEC by participating in important cancer-related biological processes. Our study will help to improve our understanding of underlying mechanisms in the progression of UCEC and provide novel lncRNAs as candidate predictive biomarkers for the identification of patients with high risk for progression.

## INTRODUCTION

Endometrial carcinoma is the most common malignancy in the female population with a rapidly increasing trend worldwide [1]. Although the outcome is favorable for many cases diagnosed at an early stage with a five-year survival rate of 75%∼86% [2], some will relapse and eventually die. Treatment of endometrial cancer is dependent on the stage of the disease and surgical intervention, if possible, is the standard management. The majority of endometrial carcinoma patients with early stage will be cured with surgery alone. Adjuvant therapy (including radiation therapy and/or chemotherapy) after surgical intervention is another treatment option in cases of high-risk or advanced endometrial carcinoma patients and has been shown to improve survival in patients with advanced stage [3]. However, the fact that a subgroup of patients with early stage faced an increased risk of cancer progression and recurrence, has led to an urgent need to identify predictive biomarkers that help clinicians determine which patients with early-stage might benefit from more aggressive therapy.

The sequencing of the human genome has suggested that only < 2% of the total genomic sequence encodes only ∼20,000 protein coding genes, whereas most of the human genome can be transcribed, yielding tens of thousands of non-coding RNAs (ncRNA) [4, 5]. NcRNAs are grouped into two major categories based on transcript size: small ncRNAs and long non-coding RNAs (lncRNAs). LncRNAs, representing the major class of ncRNAs, was arbitrarily defined as mRNA-like transcripts ranging in length from 200 nucleotides (nt) to ∼ 100 kilobases (kb) lacking significant protein-coding capacity [6]. A large number of studies have shown that lncRNAs play a critical role in various fundamental biological processes by regulating gene expression at epigenetic, transcriptional, post-transcriptional levels [7–9]. Aberrant lncRNA expression has widely been observed in various cancers [10–12]. It is becoming increasingly apparent that these dysregulated lncRNAs are specifically associated with the development and progression of cancers [13–18]. Some well-characterized lncRNAs have been found to possess oncogenic or tumor suppressive roles and function as a biomarker for cancer diagnosis or prognosis [19, 20], such as *H19*, *HOTAIR*, *MALAT-1*, *HULC* and so on. However, the lncRNA biomarkers for EC progression has not been previously explored.

In this study, we investigated lncRNA expression profiles in a large cohort of patients with uterine corpus endometrial carcinoma (UCEC) and attempted to identify lncRNAs capable of identifying patients at high risk for progression to advanced UCEC as novel clinical predictive biomarkers.

## RESULTS

### Identification of deregulated lncRNA expression during the progression of UCEC

**Figure 1:**
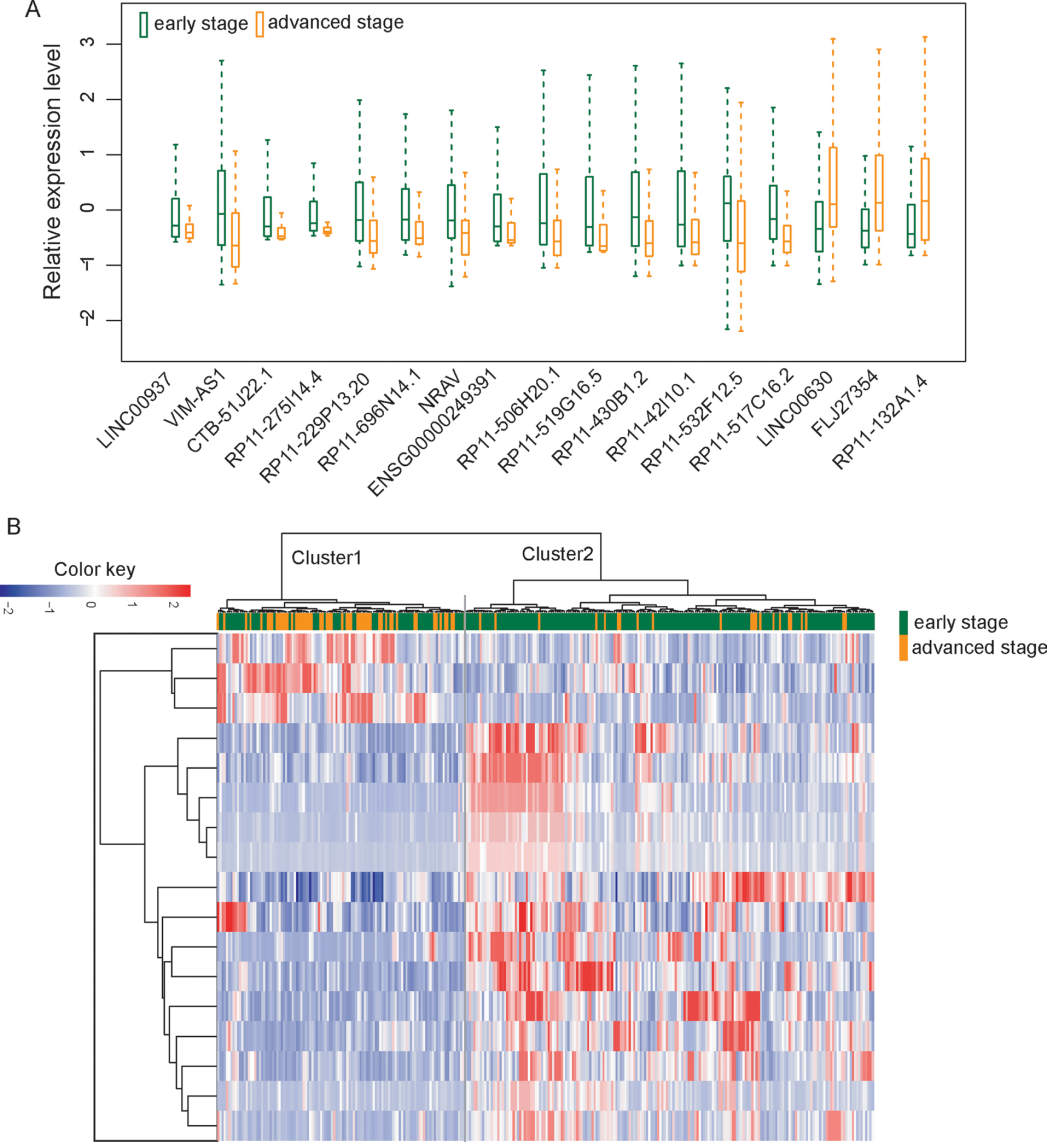
Altered lncRNA expression patterns in the progression of UCEC (**A**) Boxplots of 17 differentially expressed lncRNAs between patients with advanced-stage and those with early-stage. (**B**) The unsupervised hierarchical clustering heatmap of 300 UCEC samples based on the expression profiles of 17 differentially expressed lncRNAs

We first investigated whether there was altered lncRNA expression pattern during the progression of UCEC by comparing lncRNA expression profiles of advanced-stage patients with those with early-stage. A total of 17 lncRNAs were differentially expressed between patients with advanced-stage and those with early-stage using *T*-test with a false discovery rate (FDR) < 0.01 after Benjamini & Hochberg correction and t-statistic > 4 (or < −4) (Supplementary Table 1). Among them, 14 lncRNAs were down-regulated and three lncRNAs were up-regulated in patients with advanced stage compared with those with early stage (Figure 1A).

**Figure 2:**
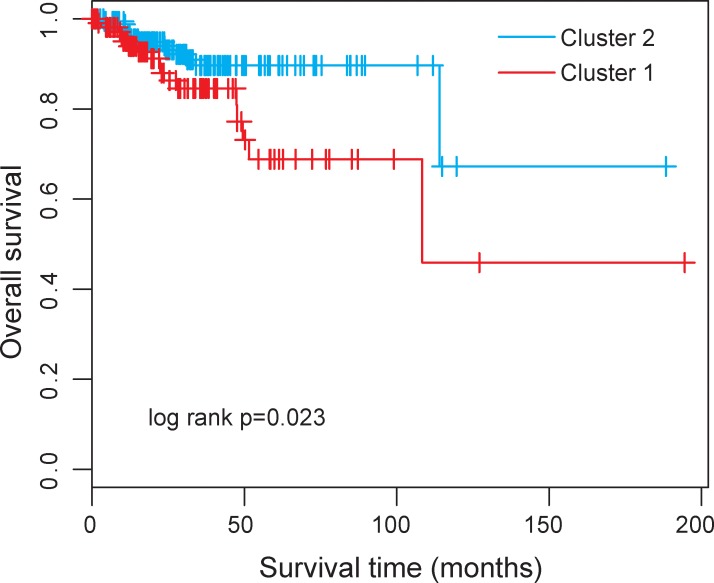
Kaplan-Meier survival curve for overall survival between the predicted two clusters based on 17 differentially expressed lncRNAs

Then we clustered 300 UCEC patients according to the expression level of differentially expressed 17 lncRNAs which resulted in two distinctive patient clusters (Figure 1B). The results of chi-square test showed that the disease progression state of the two patient clusters was significantly different (*p* < 0.001, chi-square test). Specifically, Cluster 1 contained close to the majority of advanced-stage patients (*n* = 54; 70.1%). Conversely, Cluster 2 contained the majority of early-stage patients (*n* = 164; 73.5%). The Kaplan-Meier analysis and log-rank test revealed that the overall survival time between the two patient clusters was significantly different (*p* = 0.023, log-rank test) (Figure 2). At three and five years, the survival rates of UCEC patients in Cluster 1 were 84.6% and 68.8%, respectively, whereas the corresponding rates in the Cluster 2 were 89.7% and 89.7%, respectively. The above results demonstrated that these 17 altered lncRNAs might serve as predictive biomarkers for the identification of patients with high risk for progression.

### Identification of optimal predictive lncRNA biomarkers of UCEC progression

**Figure 3:**
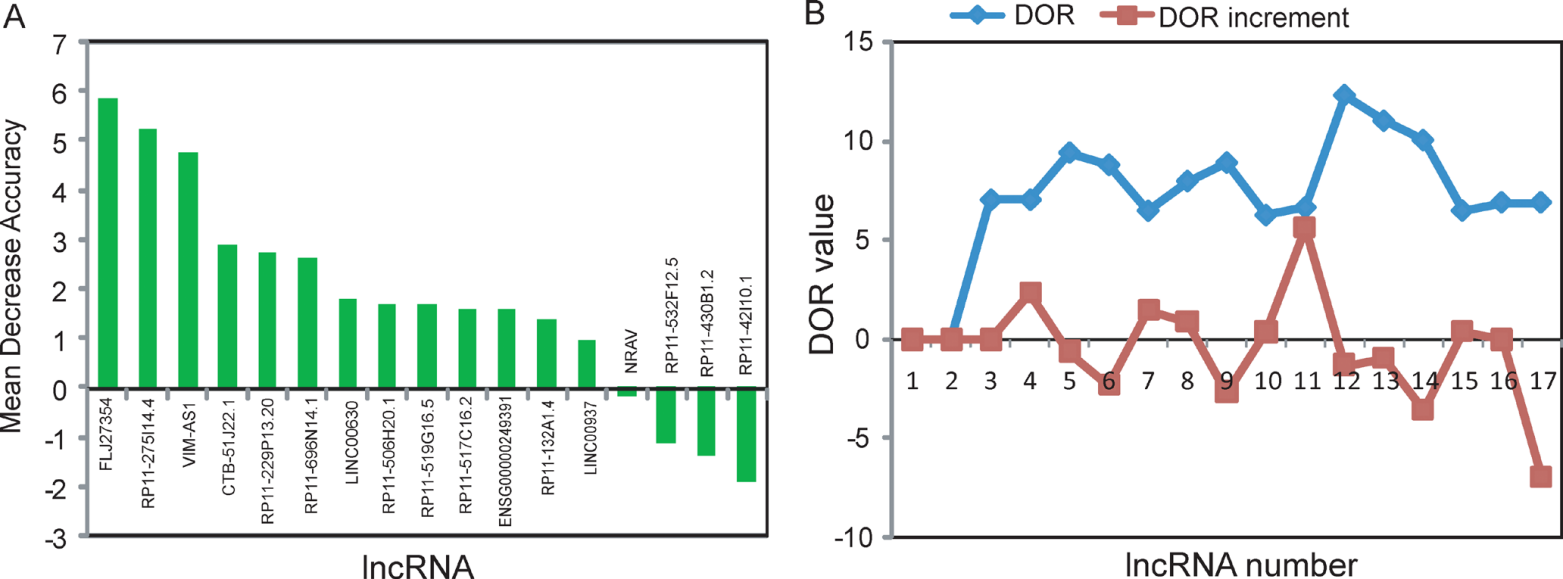
Identification of lncRNA biomarkers associated with the progression of UCEC (**A**) The variance rate of prediction accuracy for each of differentially expressed lncRNA. (**B**) The variance rate of classification performance when increasing numbers of the predictive lncRNAs.

To identify optimal predictive lncRNA biomarkers capable of identifying patients at high risk for progression to advanced stage, we performed feature selection and classification procedure using support vector machine and random forest method as described in Materials and methods. All differentially expressed lncRNAs were ranked according to the standardized drop in prediction accuracy as shown in Figure 3A. Then we compared diagnostic odds ratio (DOR) increment for a specific number of lncRNAs by subsequently adding one lncRNA at a time in a top-down forward-wrapper approach starting with the top two lncRNAs of the ranked list and identified five lncRNAs as a balance between classification accuracy and the number of lncRNAs. When choosing more than five lncRNAs, there is a downward trend in predictive performance (Figure 3B). Therefore, top five lncRNAs (*FLJ27354*, *RP11–275I14.4*, *VIM-AS1*, *CTB-51J22.1* and *RP11-229P13.20*) in the ranked list were selected as optimal predictive lncRNA biomarkers of UCEC progression (Table 1). Among them, one lncRNAs (*FLJ27354*) tended to be active and the remaining four lncRNAs (*RP11-275I14.4*, *VIM-AS1*, *CTB-51J22.1* and *RP11-229P13.20*) were silent in the progression of UCEC.

**Table 1:** Five lncRNA biomarkers associated with the progression of UCEC

Ensembl id	Gene name	Chromosomal location	t-statistic	*p*-value	FDR
ENSG00000231999.2	FLJ27354	Chr1: 89,583,241–89,632,894 (−)	4.35	3.63E-05	0.00385
ENSG00000234478.1	RP11-275I14.4	Chr1: 226,148,003–226,155,071 (+)	−4.06	6.28E-05	0.00541
ENSG00000229124.1	VIM-AS1	Chr10: 17,214,239–17,229,985 (−)	−5.04	1.19E-06	0.00041
ENSG00000232415.1	CTB-51J22.1	Chr7: 74,059,576–74,062,284 (−)	−4.3	2.39E-05	0.00274
ENSG00000235117.1	RP11–229P13.20	Chr9: 137,037,040–137,037,955 (+)	−4.53	1.01E-05	0.00175

### Performance evaluation of five lncRNA biomarkers for UCEC progression

**Figure 4:**
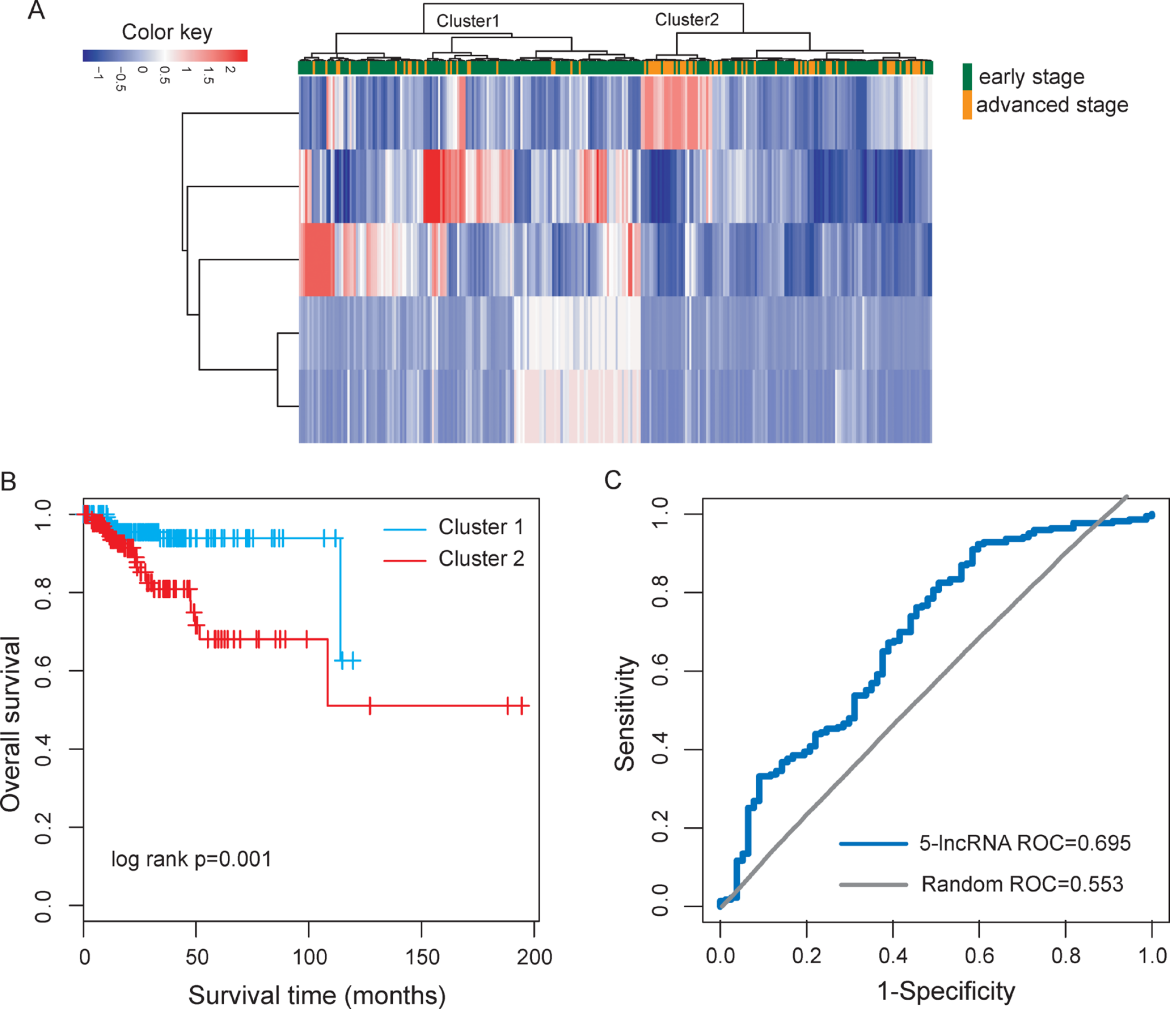
Performance evaluation of five lncRNA biomarkers in distinguishing advanced stage from early stage (**A**) The unsupervised hierarchical clustering heatmap of 300 UCEC samples based on the expression profiles of five lncRNA biomarkers. (**B**) Kaplan-Meier survival curve for overall survival between the predicted two risk groups. (**C**) ROC analysis of five-lncRNA risk classifier using LOOCV.

To test whether selected optimal five lncRNA biomarkers could efficiently distinguish high-risk patients from low-risk patients, we performed unsupervised hierarchical clustering for 300 UCEC patients according to the expression values of five lncRNA biomarkers. The results of hierarchical clustering showed that all patients were grouped into two distinctive patients clusters (162 samples in Cluster 1 vs. 138 samples in Cluster 2), which were highly correlated with disease progression status (*p* < 0.001, Fisher exact test; Figure 4A). As seen in Figure 4A, most of the advanced patients (71.4%, 55/77) were clustered into Cluster 2 and most of the early patients (62.8%, 140/223) were clustered into Cluster 1. Furthermore, the Kaplan-Meier analysis for overall survival demonstrated a significant difference between the groups predicted to be high-risk or low-risk (*p* = 0.001, log-rank test; Figure 4B). At three and five years, the survival rates of UCEC patients in the predicted high-risk group were 80.9% and 68.1%, respectively, whereas the corresponding rates in the predicted low-risk group were both 93.9%, respectively. These results revealed the better predictive performance of five lncRNAs biomarkers for the identification of patients with high risk for progression.

Thus, we integrated these five lncRNA biomarkers to construct a five-lncRNA risk classifier by using SVM algorithm. The performance of the five-lncRNA risk classifier in distinguishing advanced-stage UCEC patients from early-stage patients was evaluated in the TCGA cohort using the leave one out cross-validation (LOOCV) procedure, in which 299 patients were used as training set and the remaining one was served as the test patient. Results of LOOCV procedure showed that the five-lncRNA risk classifier for distinguishing advanced-stage patients from early-stage patients achieves 78% prediction accuracy with 96.6% sensitivity and 76.6% specificity. The discriminatory performance of the five-lncRNA risk classifier, evaluated by calculating the receiver operating characteristic curve (AUC) and DOR, revealed that the AUC was 0.695 (Figure 4C) and the DOR was 9.4. These results demonstrated that the five-lncRNA risk classifier had the better predictive performance for identifying patients at risk for UCEC progression.

### Functional implication of five lncRNA biomarkers

**Figure 5:**
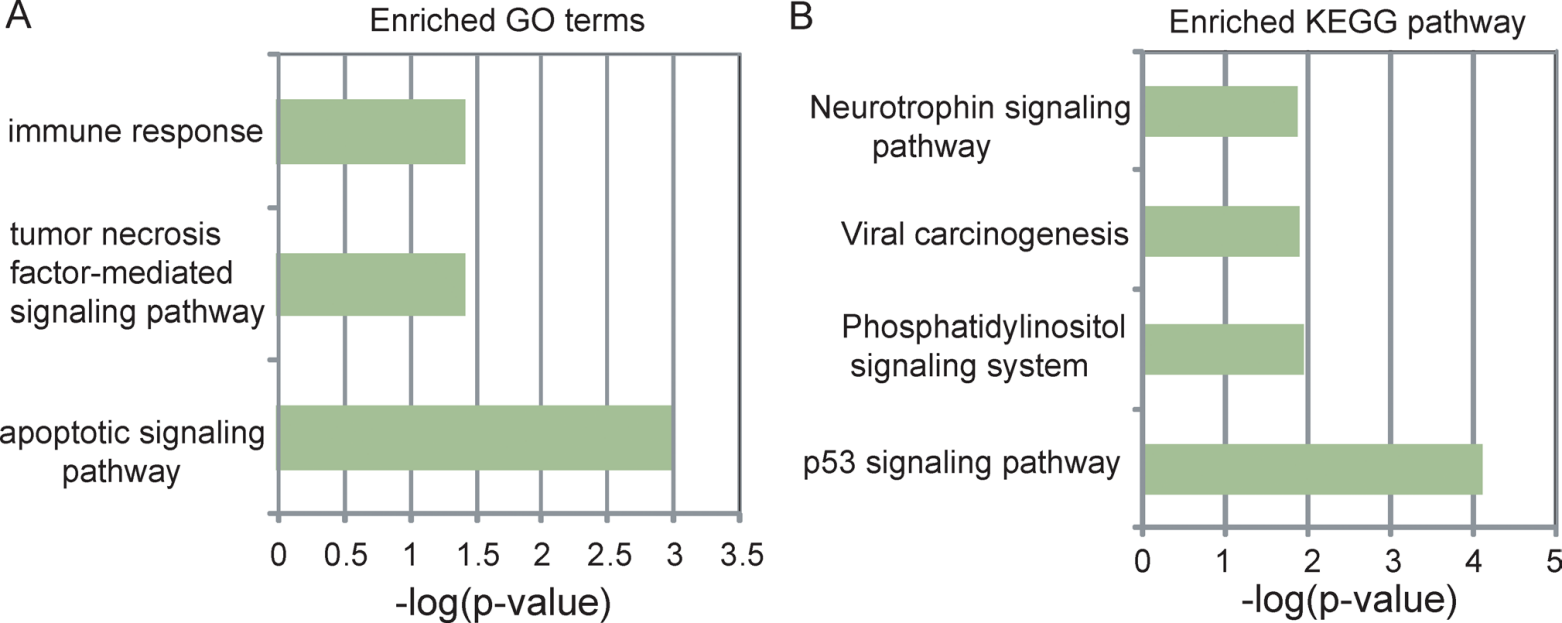
Functional prediction of five lncRNA biomarkers (**A**) The enriched GO terms ranked by–log10 (*p*-value). (**B**) The enriched KEGG pathways ranked by –log10 (*p*-value).

To explore the potential functional role of five lncRNA biomarkers in the progression of UCEC, we first examined the expression correlation between each of five lncRNA biomarkers and mRNAs in the TCGA cohort and identified 625 mRNAs correlated with at least one of the five lncRNA biomarkers (Pearson correlation coefficient > 0.5 and p < 0.01). Then we performed functional enrichment analysis of mRNAs correlated with the five lncRNA biomarkers for Gene Ontology (GO) and Kyoto Encyclopedia of Genes and Genomes (KEGG). The results of GO analysis suggested that the 625 mRNAs clustered most significantly in three GO terms (including apoptotic signaling pathway, tumor necrosis factor-mediated signaling pathway and immune response) (Figure 5A) and four KEGG pathways (including p53 signaling pathway, Phosphatidylinositol signaling system and Viral carcinogenesis, Neurotrophin signaling pathway) (Figure 5B). These enriched functional categories are well known to be associated with the development and progression of cancer. Therefore, it is a plausible inference that dysregulated expression of five lncRNA biomarkers may lead to UCEC tumorigenesis and progress via regulating mRNAs involved in the known key cancer-associated pathway.

## DISCUSSION

Endometrial carcinoma is the most common gynecologic malignancy. Standard management of endometrial cancer at diagnosis involves surgery, followed by chemotherapy with or without radiation therapy. Traditional histopathologic features, including histologic grade, tumor diameter, depth of myometrial invasion and status of lymphovascular space involvement, have been used to identify those at high-risk for disease progression and guide adjuvant treatment decisions [21]. Like many malignancies, UCEC is a complex disease characterized by both genetic, epigenetic and environmental factors [22]. The risk factors associated with disease relapse remain unclear. Because having no consideration of molecular heterogeneity, traditional histopathologic features are insufficient for making adjuvant treatment decisions [23]. Previous studies have focused on altered mRNA and miRNA expression and identified several molecular biomarkers for survival and recurrence prediction of endometrial carcinoma patients [21, 24–27]. Recently, dysregulated lncRNA expression has been implicated in the development and progression of tumors. Increasing evidence suggests that lncRNAs have an intrinsic advantage in their use as diagnostic or prognostic biomarkers compared to protein-coding genes and miRNAs since expression of lncRNA is a better indicator of the tumor status [28]. The emerging roles of lncRNAs in endometrial carcinoma have been investigated in several studies. For example, a study of 3 paired endometrial carcinoma and adjacent non-tumor tissues identified 53 differentially expressed lncRNAs and validated the potential function of lncRNA *ASLNC04080* in endometrial carcinoma genesis and progression [29]. Another study performed by Xu *et al*. also identified 172 dysregulated lncRNAs by studying the expression profiles of lncRNA in EC as compared to normal endometrium [30]. Although the above studies revealed perturbed expression of lncRNAs in endometrial carcinoma, the research of diagnostic and prognostic value of lncRNAs is presently in its infancy.

In this study, we performed genome-wide analysis of 1377 lncRNAs in a large number of endometrial carcinoma patients from TCGA and found altered lncRNA expression patterns during the progression of UCEC, implying the potential roles of lncRNA as predictive biomarkers for the discrimination of the high-risk endometrial carcinoma patients. In order to predict lncRNA biomarkers specific to endometrial carcinoma progression, we have searched for lncRNA combinations among the 17 differentially expressed lncRNAs, whose expression pattern may distinguish high-risk patients from those with early-stage using random forest feature selection which is necessary to avoid a small sample-per-feature ratio and provide better classification [31, 32]. A five-lncRNA combination, (*FLJ27354*, *RP11-275I14.4*, *VIM-AS1*, *CTB-51J22.1* and *RP11-229P13.20*), has been identified as optimal biomarkers for EC progression. Then these five lncRNAs were integrated into a risk classifier using support vector machine and achieved a 78% prediction accuracy with 96.6% sensitivity and 76.6% specificity in stratifying early and later stages of endometrial carcinoma patients using LOOCV. As demonstrated in previous studies [33, 34], LOOCV has been widely recognized and increasingly used by investigators to examine the quality of various classifiers with SVM as the prediction engine. These findings demonstrated the feasibility and potential power of the five lncRNA biomarkers in identifying endometrial carcinoma patients at high risk for progression.

Although more and more lncRNAs have been identified, current knowledge for functional roles is relatively limited and only a few of lncRNAs have been well functionally characterized. Increasing evidence has suggested that lncRNAs function by regulating or interacting with its partner molecule. Therefore, it is widely used to associate specific lncRNAs with biological processes by correlating a common expression pattern of lncRNAs with protein-coding genes [28, 35]. Inspired by the above method, in order to investigate the functional roles of identified five lncRNA biomarkers in UCEC biology, we first identified protein-coding genes that are co-expressed with each lncRNA biomarker, and then performed guilt by association analysis to identify the potential function of lncRNAs by performing functional enrichment analysis for their co-expressed protein-coding genes. According to the above analysis, five lncRNA biomarkers were predicted to participate in several known cancer-related biological progress such as p53 signaling pathway, Phosphatidylinositol signaling system and Viral carcinogenesis, Neurotrophin signaling pathway. Previous studies have shown that aberrant P53 signaling pathways might play an important role in uterine and endometrial cancer [36]. In human endometrial carcinoma, p53 mutations the most frequent genetic events identified in aggressive nonendometrioid cancer [37]. Multiple links between the cellular phosphoinositide system and cancer have been observed [38]. For UCEC, Phosphatidylinositol 3-kinase signaling regulates insulin-like growth factor binding protein-3 expression in endometrial cancer cell lines [39]. Moreover, there were important interactions between the PI3K-AKT and p53 signaling pathways [40]. Neurotrophin signaling in the pathogenesis of cancer has been found to be associated with to stimulation of mitogenesis, promotion of metastasis and invasiveness, and inhibition of apoptosis [41]. These results of guilt by association analysis suggested that these five lncRNA biomarkers may play critical roles in the progression of UCEC by participating in important cancer-related biological processes.

In conclusion, our study has shown that the lncRNA expression profiles are altered in the advanced-stage UCEC patients compared with early-stage patients. We identified five novel lncRNA biomarkers that are significantly associated with the progression of UCEC by using random forest feature selection procedure, and developed a five-lncRNA risk classifier using SVM which significantly discriminate high-risk UCEC patients from persons with early stage with high performance. To our knowledge, it is the first investigation to identify lncRNA biomarkers for UCEC progression. Further validation studies in prospective datasets are needed to test the predictive power of the risk classifier before it is applied clinically.

## MATERIALS AND METHODS

### Patient and clinical characteristics

Clinical characteristics of 300 UCEC patients with stage information were obtained from The Cancer Genome Atlas (TCGA) project (https://cancergenome.nih.gov/). UCEC patients used in this study included 223 early-stage patients (207 patients with stage I and 16 patients with stage II) and 77 advanced-stage patients (64 patients with stage III and 13 patients with stage IV). The detailed clinical characteristics of 300 UCEC patients used in this study were summarized in Table 2.

**Table 2:** Clinical covariates for the TCGA UCEC cohort

Variables		Total TCGA *N* = 300
Stage, no(%)	I	207(68.9)
	II	16(5.3)
	III	64(21.3)
	IV	13(4.3)
Grade, no(%)	1	70(23.3)
	2	80(26.7)
	3	150(50)
histology, no(%)	Endometrioid	242(80.7)
	Serous	50(16.7)
	Mixed	8(2.7)
Vital status (%)	Alive	269(89.7)
	Dead	31(10.3)
Age, years, Mean (range)		63.4(62.1–64.6)

### Genome-wide RNA-sequencing data of mRNAs and lncRNAs in UCEC patients

Genome-wide lncRNA and mRNA expression of 300 UCEC patients were retrieved from TCGA long non-coding RNAs database (http://larssonlab.org/tcga-lncrnas/index.php) according to Akrami *et al*.[42], including 10419 lncRNAs and 15977 mRNAs, respectively. Briefly, RNA-seq data of TCGA UCEC patients in BAM format were realigned to the Hg19 assembly with TopHat and read counts for individual GENCODE genes were subsequently determined using HTSeq-count in “intersection-strict” mode, by considering only uniquely mapped reads. RPKM expression levels for lncRNAs and mRNAs were finally calculated by normalizing for lncRNA and mRNA length, and were log2 transformed. Then those lncRNAs with missing expression values in >10% samples were filtered which resulted in 1377 lncRNAs for subsequent analysis.

### Analysis of lncRNA expression profiles

Differential expression analysis by comparing lncRNA expression pattern in early-stage patients with those in advanced-stage patients using student *t*-test based on log-scale expression values. Differentially expressed lncRNAs were identified at the threshold of t-statistic > 4 (or < −4) and false discovery rate (FDR) < 0.01 (Benjamini and Hochberg algorithm). Hierarchical clustering of the expression values of differentially expressed lncRNAs was performed with R package “pheatmap” using the metric of Euclidean distance and complete linkage. The chi-square test was used to evaluate the significance between disease progression status and lncRNA biomarkers.

### Statistics for classification and prediction

For classification of early-stage patients vs. advanced-stage patients, a support vector machine (SVM) was applied with the sigmoid kernel using R package “randomForest”. An unbiased performance estimate in the classification of early-stage patients vs. advanced-stage patients was performed using leave one out cross-validation (LOOCV). Diagnostic ability of classification prediction was evaluated by obtaining the area under a receiver operating characteristic (ROC) curve (AUC) and diagnostic odds ratio (DOR). Kaplan-Meier survival plots and log-rank tests were used to assess the differences in patient outcomes between the predicted high-risk and low-risk groups.

To identify optimal lncRNA biomarkers stratifying early and advanced stages of UCEC, we performed feature selection procedure as previously described [31]: (i) random forest importance value for each of differentially expressed lncRNA were obtained to represent the standardized drop in prediction accuracy. (ii) differentially expressed lncRNAs was re-ranked according to their random forest importance value. (iii) finding the optimal number of features by subsequently adding one lncRNA at a time in a top down forward-wrapper approach starting with the top two lncRNAs of the ranked list; at each increment, the DOR was assessed using LOOCV.

### Functional enrichment analysis

Functional enrichment analysis of GO and KEGG was performed using DAVID Bioinformatics Tool (version 6.7) [72] limited to GO terms in the “Biological Process”(GOTERM-BP-FAT) and KEGG pathway categories. The biological processes and pathways with *p*-value of < 0.05 using the whole human genome as background were considered as significant enriched functional categories.

## SUPPLEMENTARY MATERIALS TABLE


