# Supplementary material for: Identification of long non-coding RNAs biomarkers associated with progression of endometrial carcinoma and patient outcomes
Source: Oncotarget. 2017 Apr 30;8(32):52604–13. doi: 10.18632/oncotarget.17537 (PMC5581054; doi:10.18632/oncotarget.17537)
Supplement: Supplementary file 1 [file oncotarget-08-52604-s001.pdf]

Identification of long non-coding RNAs biomarkers associated with progression of endometrial carcinoma and patient outcomes

Supplementary Materials

Supplementary Table 1: Differentially expressed lncRNAs between patients with advanced-stage and those with early-stage

Gene ensembl	Gene symbol	Chromosome	t-statistic	*p*-value	FDR
ENSG00000259577.1	RP11-430B1.2	Chromosome 15: 52,180,093-52,205,874 forward strand	-5.97022712	8.29E-09	1.14E-05
ENSG00000246090.2	RP11-696N14.1	Chromosome 4: 99,088,857-99,301,356 forward strand	-5.245587526	2.99E-07	0.00020613
ENSG00000229124.1	VIM-AS1	Chromosome 10: 17,214,239-17,229,985 reverse strand	-5.040942818	1.19E-06	0.000409181
ENSG00000261286.1	RP11-517C16.2	Chromosome 16: 84,459,259-84,467,361 reverse strand	-5.058094222	9.56E-07	0.000409181
ENSG00000259342.1	RP11-519G16.5	Chromosome 15: 45,430,652-45,441,808 reverse strand	-4.886105306	1.98E-06	0.000544658
ENSG00000226091.2	LINC00937	Chromosome 12: 8,295,986-8,396,803 reverse strand	-4.71951332	3.66E-06	0.000719542
ENSG00000251307.1	RP11-506H20.1	Chromosome 5: 55,233,934-55,295,201 forward strand.	-4.776965662	3.34E-06	0.000719542
ENSG00000235117.1	RP11-229P13.20	Chromosome 9: 137,037,040-137,037,955 forward strand	-4.526005513	1.01E-05	0.001745813
ENSG00000223546.2	LINC00630	Chromosome X: 102,769,161-102,885,406 forward strand	4.445675577	2.32E-05	0.002740605
ENSG00000232415.1	CTB-51J22.1	Chromosome 7: 74,059,576-74,062,284 reverse strand	-4.297525635	2.39E-05	0.002740605
ENSG00000249391.1			-4.310130746	2.31E-05	0.002740605
ENSG00000261183.1	RP11-532F12.5	Chromosome 15: 40,835,808-40,844,387 reverse strand	-4.434230998	1.93E-05	0.002740605
ENSG00000231999.2	FLJ27354	Chromosome 1: 89,583,241-89,632,894 reverse strand	4.345486403	3.63E-05	0.00384575
ENSG00000260086.1	RP11-42I10.1	Chromosome 16: 48,623,436-48,744,921 forward strand	-4.195197905	4.43E-05	0.004354762
ENSG00000248008.2	NRAV	Chromosome 12: 120,490,328-120,495,940 reverse strand	-4.125418375	5.47E-05	0.00502192
ENSG00000234478.1	RP11-275I14.4	Chromosome 1: 226,148,003-226,155,071 forward strand	-4.060215377	6.28E-05	0.005405939
ENSG00000232445.1	RP11-132A1.4	Chromosome 7: 101,308,346-101,310,985 forward strand	4.040902824	0.000112089	0.009079239
